# Rational Use of Bethanechol in Dogs and Cats with Bladder Dysfunction

**DOI:** 10.3390/vetsci12090918

**Published:** 2025-09-22

**Authors:** Franco Galluzzi, Alessandro Menozzi, Roberta Saleri, Fabio De Rensis, Giliola Spattini

**Affiliations:** 1 Castellarano Veterinary Clinic, 42014 Castellarano, Italy; francogall@live.it (F.G.); giliolavet@gmail.com (G.S.); 2 Department of Veterinary Science, University of Parma, 43126 Parma, Italy; roberta.saleri@unipr.it (R.S.); fabio.derensis@unipr.it (F.D.R.)

**Keywords:** bethanechol, urinary retention, dog, cat, bladder dysfunction, muscarinic agonist

## Abstract

Bethanechol chloride, a nonselective muscarinic agonist, is the most frequently employed drug in dogs and cats to induce detrusor smooth muscle contraction under conditions characterized by poor or absent bladder emptying. Although this drug has minimal or absent nicotinic activity, at higher doses, weak stimulation of neuronal nicotinic receptors may occur, causing the release of noradrenaline, which induces contraction of the urethral smooth muscle by activating α-adrenergic receptors. In the presence of total or partial suprasacral lesions, the elaboration and initiation phase of the urination process is absent due to an interruption of afferent signals from the bladder to the brainstem. In such cases, hypertonicity of the urethral sphincters is expected, and bethanechol is contraindicated. Bethanechol is also not indicated for reflex dyssynergia. In the presence of complete injuries involving the sacral segments, cauda equina, or pelvic nerve, both reflex and voluntary micturition are abolished, and bethanechol is usually ineffective. However, in cases of partial injuries, bethanechol is likely to be effective, as partial integrity of the micturition reflex is required to produce sustained bladder contraction. Bethanechol may benefit patients with myopathic decompensated bladder, although its effectiveness depends on the severity of detrusor damage.

## Simple Summary

In dogs and cats with bladder voiding deficit, pharmacological treatment mostly involves the use of the muscarinic agonist bethanechol, even though the use of this drug remains controversial. Clinical and experimental studies in humans and animals seem to indicate that bethanechol may be effective only depending on the nature of bladder dysfunction, and that a thorough evaluation of the pathologic condition is mandatory to assess if the administration of this drug is indicated. In the presence of total or partial lesions above sacral spinal segment, the propagation of afferent signals from the bladder to the brainstem is interrupted; therefore, the elaboration and initiation phase of the urination process is missing. In this condition, the smooth muscle of urethral sphincters is likely to be contracted, and bethanechol is therefore contraindicated. Complete injuries involving the sacral segments, cauda equina, or pelvic nerve cause an absence of both reflex and voluntary micturition, and in these patients, bethanechol is usually ineffective. By contrast, in cases of incomplete injuries, bethanechol is likely to be effective, as there is evidence that partial integrity of the micturition reflex is essential to produce a sustained bladder contraction. Bethanechol is contraindicated in patients with reflex dyssynergia as it may worsen bladder/urethral incoordination. Conversely, bethanechol may benefit patients with myopathic decompensated bladder, although the severity of detrusor damage deeply influences its efficacy.

## 1. Introduction

Bladder emptying requires a well-coordinated detrusor contraction of adequate magnitude, a concomitant lowering of outflow resistance at the level of the smooth and striated sphincters, and the absence of anatomic obstruction. If these requirements are not fulfilled, urinary retention will result. Detrusor hypo/acontractility may be caused by neurological impairment or a smooth muscle functional disorder. Since the autonomic nervous system regulates bladder smooth muscle motility, and parasympathetic innervation is crucial for detrusor muscle contraction, cholinergic drugs such as muscarinic agonists have been extensively used to promote bladder emptying [1].

Bethanechol chloride, a nonselective muscarinic agonist, is the most frequently employed drug in human patients, as well as in dogs and cats, to induce detrusor smooth muscle contraction under conditions with poor or absent bladder emptying. Although other cholinomimetic drugs have been synthesized and some tested for clinical use, none have demonstrated clear advantages over bethanechol.

In human medicine, despite its widespread clinical use, bethanechol administration has not always resulted in improved clinical outcomes in cases of urinary retention, and the effectiveness of parasympathomimetic agents in underactive bladder has recently been called into question [2]. In dogs and cats, bethanechol has been utilized for many years, but the results remain equivocal [3,4,5,6].

The present review aims to shed light on the mechanism of action and clinical data regarding the use of bethanechol in bladder-emptying dysfunctions, and to contribute to a more rational use of this drug in dogs and cats, since a thorough evaluation of the indications for the use of this drug in these species is at present lacking.

This is a narrative review therefore the authors did not perform a systematic review of the available literature; since the bibliography on bethanechol use in dogs and cats with bladder dysfunctions is scarce, the inclusion criteria for the references focused primarily on the relevance of the studies to the specific topic. When a specific reference in dogs and cats was not available, studies in human patients published in important scientific journals were cited, and this may represent a limitation of this review.

## 2. Anatomical and Physiological Basis of Micturition

**Figure 1:**
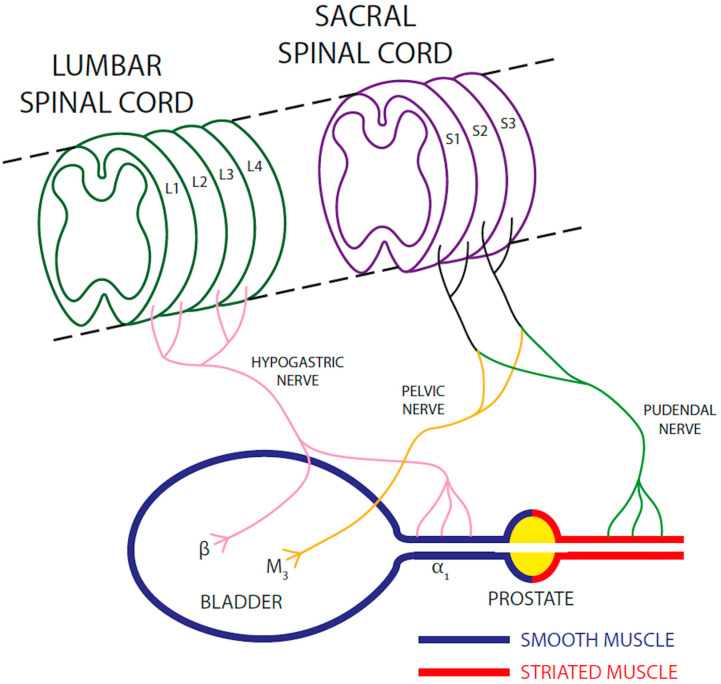
Innervation of the bladder and urethral sphincters. L1–L4: lumbar spine segments; S1–S3: sacral spine segments; β_3_: β_3_-adrenergic receptor; α_1_ = α_1_-adrenergic receptor; M_3_ = M_3_ muscarinic receptor.

Normal micturition, the mechanism through which animals expel urine from the body, requires perfect coordination between bladder smooth muscle contraction and relaxation of the urethral sphincters. The innervation of the bladder and urethral sphincters is described in Figure 1.

The micturition process comprises two distinct phases: storage and voiding. During the storage phase, continence must be ensured; thus, the sympathetic nervous system maintains the tone of the proximal urethra via the hypogastric nerve. The smooth muscle of this urethral tract contains an abundant population of α_1_-adrenergic receptors which, when activated by noradrenaline released from sympathetic neurons, contract the urethra and prevent urine outflow from the bladder. Simultaneously, noradrenaline released by the hypogastric nerve relaxes the detrusor muscle by acting on β-adrenergic receptors, allowing bladder dilation necessary for the filling process.

In this phase, somatic innervation is also involved: acetylcholine released from the pudendal nerve stimulates contraction of the striated muscle in the distal portion of the urethra, further contributing to the inhibition of urine outflow. During the storage phase, the pelvic parasympathetic nerve is not activated, and therefore the detrusor muscle remains relaxed.

Throughout the voiding phase, the hypogastric (sympathetic) and pudendal (somatic) nerves are inhibited, while the pelvic (parasympathetic) nerve is activated, releasing acetylcholine that by activating muscarinic receptors induces detrusor muscle contraction and bladder emptying. In both phases, the cerebral cortex receives signals from the urinary tract—interpreted as a “full” or “empty” bladder—and sends back signals that modulate the micturition process accordingly.

In dogs and cats, cholinergic and adrenergic receptors have a similar distribution in the bladder and urethra [7,8].

## 3. Etiopathogenesis of Detrusor Contraction Deficit

A decrease in detrusor muscle contractility can often lead to partial or total urinary retention, and impairment of the bladder emptying mechanism may be of neurogenic or myogenic origin. Neurogenic emptying dysfunction is usually caused by damage to afferent/efferent innervation or to the central nervous system. Impairment of neuronal pathways leads to reduced acetylcholine release from parasympathetic nerve terminals (pelvic nerve) and/or enhanced degradation of the neurotransmitter by the acetylcholinesterase enzyme, resulting in insufficient activation of muscarinic receptors in detrusor smooth muscle [9].

In cases of bladder-emptying dysfunction of myogenic origin, patients are neurologically normal, but the smooth muscle is damaged and unable to contract effectively. This can occur due to aging, chronic inflammation, or long-lasting outlet obstruction.

## 4. Pharmacological Properties of Bethanechol

Bethanechol is approved by the Food and Drug Administration (FDA) in the United States to treat postoperative and postpartum urinary retention or neurogenic atony of the bladder in human patients [10] and was authorized by European Medicines Agency in most European countries against bladder atony following surgery. In Italy, bethanechol is currently available only as a galenical preparation. Bethanechol (carbamyl-β-methylcholine) is a carbamate choline ester with significant acetylcholine-like activity; it exerts its effects by directly stimulating the muscarinic receptors of the parasympathetic nervous system [11]. Bethanechol is a nonselective muscarinic agonist, as it activates all five muscarinic receptor subtypes (M_1_, M_2_, M_3_, M_4_, and M_5_) [12]. The effects of this drug on bladder smooth muscle are mainly due to stimulation of M_3_ receptors, which are coupled to Gq proteins and induce the release of Ca^2+^ from intracellular stores, promoting contraction of the detrusor muscle necessary to initiate micturition and bladder emptying [13].

However, it appears that the action of bethanechol is not exclusively due to receptor stimulation; in fact, some studies have provided direct evidence that at least partial integrity of the micturition reflex is essential to produce sustained bladder contraction with bethanechol [14,15,16,17].

Bethanechol is highly selective for muscarinic receptors, as smooth muscle contractions induced by this drug are blocked by the muscarinic antagonist hyoscine but are not affected by the ganglion blocker hexamethonium [18]. Moreover, pre-treatment with atropine abolishes bethanechol’s pressor activity in cats and dogs [19,20]. For these reasons, bethanechol is preferred over other muscarinic agonists such as carbachol, which possesses significant nicotinic activity. However, at higher doses, weak stimulation of neuronal nicotinic receptors by bethanechol cannot be excluded, which may induce the release of noradrenaline from sympathetic nerve terminals (hypogastric nerve), causing contraction of proximal urethral smooth muscle through activation of α-adrenergic receptors [21,22].

Being a carbamate, bethanechol resists hydrolysis by acetylcholinesterase and therefore has a prolonged action [10]. Moreover, bethanechol is unable to cross the blood–brain barrier and thus does not cause central nervous system side effects [10].

Clinical data regarding bethanechol in human patients are largely derived from experimental studies conducted on animals, especially dogs, cats, and rabbits.

## 5. Effects of Bethanechol on the Bladder

In vitro studies have shown that bethanechol induces contraction of dog bladder strips by activating muscarinic receptors [21,23,24,25,26]. In vivo, an increase in intravesical pressure following bethanechol administration in dogs—both before and after experimental induction of paraplegia—has been reported, suggesting potential utility of this drug in animals with spinal shock, even though bladder emptying was partially hindered by increased urethral pressure [25].

In cats, subcutaneous administration of bethanechol has been shown to enhance spontaneous bladder activity and induce sustained contraction of the detrusor muscle. This effect was abolished in cats following spinal transection or complete sacral rhizotomy [14,15,17]. These findings support the conclusion that an intact pelvic (parasympathetic) reflex is necessary for bethanechol-induced sustained bladder contraction [14,15,17,27].

In experimental cats, failure of bethanechol to induce bladder emptying after complete sacral rhizotomy was observed in both short-term (two to three weeks) and long-term (ten weeks) experiments [27]. However, in cases of partial cauda equina lesions, the detrusor reflex may be preserved, and bethanechol could therefore be effective [27].

## 6. Effects of Bethanechol on the Urethra

Bethanechol administration causes an increase in urethral pressure in the proximal tract of the urethra, which contains smooth musculature in both dogs and cats [21,25,28,29,30]. Bethanechol is a weak agonist of cholinergic nicotinic receptors located in the ganglia of the sympathetic nervous system and may therefore evoke the release of noradrenaline, which in turn can activate α_1_-adrenergic receptors, causing contraction of the urethral smooth muscle [22].

## 7. Indications for a Rational Use of Bethanechol

**Figure 2:**
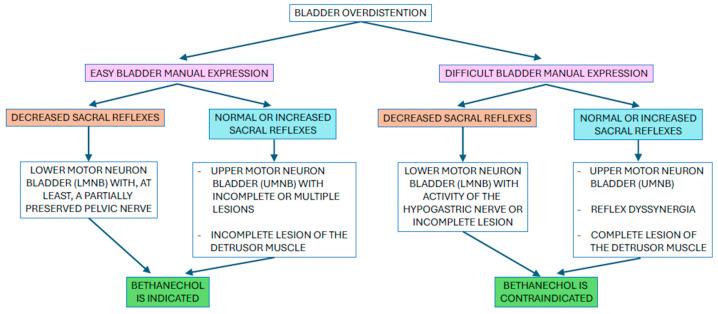
Schematic algorithm to evaluate the indication of bethanechol administration in bladder voiding dysfunctions according to clinical signs and suspected diagnosis.

Available clinical and experimental data suggest a cautious and well-considered use of bethanechol, tailored to the type of bladder dysfunction involved, while avoiding indiscriminate administration of the drug. Bladder outlet obstruction represents an absolute contraindication for bethanechol administration (Figure 2).

When there is a spinal injury proximal to the sacral segment, an overdistended urinary bladder is expected due to an areflexic bladder and contracted sphincters; this condition is referred to as upper motor neuron bladder (UMNB). In contrast, injury to either the sacral cord or the cauda equina segment typically results in lower motor neuron bladder (LMNB). In human medicine, studies have noted an imprecise correlation between somatic neurologic findings and characteristic urodynamic patterns [31,32,33,34]. Several factors may contribute to this discrepancy. First, reorganization of crucial neural pathways (neuroplasticity) distal to the lesion may influence both neurologic and urodynamic findings. Second, the lesion may be incomplete, thereby partially preserving the integration and modulation of complex micturition signals at multiple levels of the nervous system. Additionally, multiple injuries coexisting at different levels can result in unpredictable mixed voiding dysfunctions [34].

If there is a complete lesion of a nerve or nervous segment, urinary changes are usually consistent, and the expected clinical picture can be predicted. However, lesions are frequently incomplete and/or affect more than one neural tract, making it difficult to anticipate the clinical and functional presentation—especially due to neuroplasticity phenomena [31,32,33,34].

### 7.1. Total or Partial Suprasacral Lesions (UMNB)

In these cases, the lesion interrupts the propagation of afferent signals from the bladder to the brainstem, thereby blocking the elaboration and initiation phase of the urination process. Initially, an areflexic bladder with complete urinary retention occurs due to the failure of the pons (micturition center) to activate the urination process. Subsequently, “automatic micturition” develops, mediated by sacral spinal reflexes, so that urination becomes involuntary [35].

In particular, the sacral tract—no longer in communication with the pons—becomes autonomous and activates detrusor contraction (via the pelvic nerve) independently (reflex). As the bladder fills, the increase in intravesical pressure triggers a reflex contraction, resulting in involuntary urination due to detrusor instability. At the same time, because of the lack of pontine activation of the micturition reflex, relaxation of the urethral sphincters does not occur. Thus, when the bladder contracts, the sphincters remain closed, preventing bladder emptying and leading to urinary incontinence due to bladder overdistension.

On physical examination, the bladder appears distended but is difficult to empty manually, as the urethral sphincters remain tonic and fail to relax [36]. Sacral reflexes remain normal or are increased. Among the most common causes of this condition are herniated disks, fractures, vertebral dislocations or subluxations, and vascular, infectious, degenerative, or neoplastic pathologies [37].

In this condition, it is essential to exclude the presence of a hypertonic urethral sphincter before considering the administration of bethanechol, in order to avoid worsening functional urethral obstruction, which would further hinder bladder emptying and could lead to damage to the bladder and kidneys [16,38].

The lack of instruments for urodynamic studies in dogs and cats—compared to human medicine—makes it difficult to assess urethral sphincter pressure and determine whether bethanechol is indicated. For these reasons, from a practical standpoint, bethanechol must not be used in dogs and cats with UMNB, due to increased urethral sphincter tone, except in cases where manual bladder voiding occurs easily, such as when incomplete or multiple lesions are present, or when neuroplasticity leads to partial restoration of nerve function over time.

### 7.2. Total or Partial Injuries Involving Sacral Segments, Cauda Equina, or the Pelvic Nerve (LMNB)

Traumatic damage and post-surgical lesions are among the most common causes of LMNB. In LMNB patients, both reflex and voluntary micturition are abolished. This condition is characterized by hypo/areflexia of the bladder due to lesions of the pelvic nerve (which controls bladder contraction) and dysfunction of the striated urethral sphincter due to lesions of the pudendal nerve (which controls the tone of the striated urethral sphincter). Urinary incontinence may occur due to bladder overdistention and the absence of somatic pudendal nerve input. Sacral reflexes (anal, perineal, and bulbocavernosus reflexes) are usually weak or absent. On physical examination, the bladder appears large and flaccid and can be easily emptied by manual compression. In certain cases, however, in both dogs and cats, sphincter tone may be preserved, making manual bladder emptying more difficult [39].

The exact pathogenetic mechanism responsible for this dysfunction remains unclear. It has been hypothesized that the hypogastric nerve, if intact, maintains the tone of the smooth muscle of proximal urethra, or that in cases of incomplete lesions, the pudendal nerve—responsible for the tone of the striated urethra—remains functional [34,39,40].

Bethanechol is usually ineffective in LMNB with complete injuries, whereas it is likely to be effective in patients with partial lesions, as at least partial integrity of the pelvic nerves appears essential for the muscarinic agonist to exert its effects [14,15,17,27]. From a practical standpoint, the use of bethanechol in LMNB may be indicated if two essential conditions are met:

The patient has low urethral resistance, which—if urodynamic studies are unavailable—can be inferred from the bladder emptying easily by manual compression.

The lesion has spared the pelvic nerves at least partially, since, as previously mentioned, pelvic nerve functionality is essential for bethanechol to be effective. This situation can be difficult to interpret clinically, but if sacral reflexes are not completely absent, it may be assumed that innervation is at least partially preserved.

### 7.3. Reflex Dyssynergia

This micturition reflex disorder is due to a lack of urethral sphincter relaxation when the bladder begins its contraction. The etiopathogenesis of this condition, as well as the location of the lesion, remains unclear. A partial lesion is suspected in the reticulospinal tract, Onuf’s nucleus, or the caudal mesenteric ganglion, and it is suggested that the lesion may cause a loss of inhibitory signals to the pudendal or hypogastric nerves [41]. The deficit may involve the smooth or striated urethral sphincter, or affect both. Moreover, a local lesion of the nerves or of the smooth or striated urethral muscle cannot be ruled out [41].

This dysfunction primarily affects middle-aged, large, and giant-breed male dogs, but occasionally also female dogs and cats [42,43]. Animals affected by reflex dyssynergia begin urination with a normal urinary stream, followed immediately by short spurts, after which the urinary flow stops completely. The patient continues to strain to urinate but is unable to fully empty the bladder, resulting in a high postvoid residual volume.

In this condition, the perineal reflex is often hyperactive, suggesting that detrusor contraction is present, but the sphincters are not relaxed. Bethanechol is not indicated for the treatment of reflex dyssynergia, as it appears to aggravate detrusor–sphincter incoordination [44].

### 7.4. Myopathic Decompensated Bladder

Chronic inflammation, chronic partial urethral obstruction, and senescence are the main causes of bladder fibrosis with detrusor hypocontractility [45,46]. In cases of urinary retention, chronic bladder overdistension triggers a chain reaction that damages the urothelium and detrusor muscle, leading to ischemia, changes in the extracellular matrix, and detachment of the tight junctions [40,41].

Under this condition, the problem lies in the inability of the detrusor smooth muscle to produce an effective contraction to empty the bladder, despite relaxation of the urethral sphincters during attempted voiding. Bethanechol may be beneficial in these patients, although the severity of detrusor muscle damage can significantly influence the efficacy of the treatment [38].

Physical examination typically reveals a large, distended bladder. Manual bladder expression may be difficult due to normal sphincter tone. The animal may be able to partially empty the bladder through abdominal contraction. Sacral reflexes are usually intact in this condition.

In this dysfunction, as in those previously described, it is mandatory to evaluate urethral resistance before using bethanechol. This can be achieved by testing the degree of difficulty in obtaining urinary flow through manual bladder expression.

## 8. Bethanechol Dosage and Route of Administration

Bethanechol may be administered subcutaneously or orally. When given orally, its effects take longer to occur but last longer compared to parenteral administration. By contrast, subcutaneous administration appears to induce a quicker, shorter, but greater increase in intravesical pressure compared to oral administration [47].

In human patients, an initial subcutaneous administration of bethanechol is recommended, followed by oral administration once an improvement in clinical signs is observed [48]. It is also advisable to start with a low-to-medium dose of the drug, and then progressively increase or decrease the dose depending on the patient’s response. Once reflex bladder emptying is fully re-established, the drug may be withdrawn, and the patient should be examined periodically to ensure that regular, complete bladder emptying is maintained [38].

Unfortunately, in Italy, registered bethanechol formulations are no longer available; only galenical formulations for oral administration can be used.

The recommended dose of bethanechol in dogs is 2.5–15 mg orally or by subcutaneous injection every 8 h. In cats, a much lower dosage is usually employed and is indicated only for oral administration (1.25–5 mg every 8–12 h) [43,49].

## 9. Bethanechol Side Effects

Since muscarinic receptors are present in most tissues throughout the body, and bethanechol is a non-selective muscarinic agonist, several side effects may occur following drug administration. The most frequently observed side effects after bethanechol administration include anorexia, hypersalivation, vomiting, diarrhea, and bronchospasm [36,45]. Furthermore, severe cardiovascular depression, which could lead to cardiocirculatory failure, has been reported as a very rare but potentially life-threatening side effect [45].

Side effects appear to be milder after oral administration of the drug compared to those observed following subcutaneous administration [50]. In some patients, bethanechol fails to improve bladder voiding, possibly due to contraction of the urethral muscle. Combined administration of bethanechol and drugs with α-adrenergic blocking effects (e.g., tamsulosin, phenoxybenzamine) may help overcome urethral pressure, allowing effective bladder emptying [22].

## 10. Conclusions

Based on current recommendations, neurogenic bladder, acute postoperative, and postpartum functional urinary retention are acceptable indications for the use of bethanechol in human patients. Although bethanechol has been widely used in human urology, many clinicians currently consider it ineffective for most neurologic etiologies of bladder dysfunction [38,51,52].

In veterinary medicine, bethanechol has been used extensively for bladder dysfunctions characterized by urinary retention, mainly in dogs and cats. However, a critical and thorough evaluation of the actual evidence regarding its efficacy has not yet been performed. Accurate identification of the nature of the bladder dysfunction is a mandatory step in screening animals that could benefit from bethanechol administration, and to avoid indiscriminate use of the drug in poorly selected patients, which may risk worsening the clinical condition.

In cases of upper motor neuron bladder (UMNB), bethanechol should not be used due to the presence of tone in both the internal and external muscular sphincters. In contrast, in cases of lower motor neuron bladder (LMNB), bethanechol can be effective in inducing bladder voiding. However, before use, it is essential to determine whether tone is present in the internal and external sphincters. If tone is present, bethanechol should not be administered.

Future studies would be helpful to evaluate whether the combined administration of bethanechol with an α_1_-adrenergic antagonist, such as tamsulosin, could promote the relaxation of the urethral smooth muscle and facilitate bladder emptying in these patients.

## Data Availability

No new data were created or analyzed in this study.
